# Superior Gas Barrier Properties of Biodegradable PBST vs. PBAT Copolyesters: A Comparative Study

**DOI:** 10.3390/polym13193449

**Published:** 2021-10-08

**Authors:** Pengkai Qin, Linbo Wu, Bogeng Li, Naixiang Li, Xiaohu Pan, Junming Dai

**Affiliations:** 1 Key Laboratory of Biomass Chemical Engineering of Ministry of Education, College of Chemical and Biological Engineering, Zhejiang University, 38 Zheda Road, Hangzhou 310027, China; qinpk@zju.edu.cn; 2 State Key Laboratory of Chemical Engineering at ZJU, College of Chemical and Biological Engineering, Zhejiang University, 38 Zheda Road, Hangzhou 310027, China; bgli@zju.edu.cn; 3 Sinopec Research Institute of Yizheng Chemical Fiber Co., Ltd., Puxi Road, Yizheng 211900, China; linx.yzhx@sinopec.com (N.L.); panxh.yzhx@sinopec.com (X.P.); daijm.yzhx@sinopec.com (J.D.)

**Keywords:** biodegradable polymers, biobased polymers, aliphatic-aromatic copolyesters, poly(butylene succinate-co-terephthalate), gas barrier property

## Abstract

As a bio-based counterpart of poly(butylene adipate-co-terephthalate) (PBAT), the well-known commercially available biodegradable aliphatic-aromatic copolyester, poly(butylene succinate-co-terephthalate) (PBST) has comparable physical and mechanical properties, but its gas barrier properties, which are very important for packaging material and mulch film applications, have not yet been reported in literature. In this paper, the O_2_, CO_2_ and water vapor barrier properties of PBST vs. PBAT were comparatively studied and reported for the first time. Theoretical calculation of O_2_ and CO_2_ permeation coefficients via group contribution method was also conducted. The barrier properties of PBST show clear copolymer composition dependence due to different contribution of BS and BT repeat units and composition-dependent crystallinity. Comparing with PBAT, PBST with close copolymer and three-phase (crystalline, amorphous, rigid amorphous) compositions shows 3.5 times O_2_ and CO_2_ and 1.5 times water vapor barrier properties. The slower segment movement and less free volume of PBST, and therefore slower gas diffusion in PBST, accounts for its superior O_2_ and CO_2_ barrier, while the better hydrophilicity of PBST counteracts partial contribution of slower segment movement so that the improvement in water vapor barrier is not as high as in O_2_ and CO_2_ barrier.

## 1. Introduction

Poly(butylene adipate-co-butylene terephthalate) (PBAT) is an aliphatic-aromatic copolyester synthesized from butane diol, adipic acid and terephthalic acid. It combines the biodegradability of aliphatic polyester poly(butylene adipate) and the thermal and mechanical properties of aromatic polyester poly(butylene terephthalate). Due to acceptable comprehensive properties and relatively low cost of raw materials, PBAT became the first commercialized biodegradable copolyester. It was first commercialized by BASF with the trademark Ecoflex^®^ [1] about twenty years ago. After R&D for several decades, PBAT has become the most important flexible biodegradable polymers and find wide applications in film products, including shopping bags and mulch films.

On the other hand, PBAT still has its inherent shortcomings in physical and mechanical properties, including low Young’s modulus and gas barrier properties. These shortcomings limit its applications, especially for demanding ones. For example, agricultural mulch films require a high water vapor barrier to preserve soil moisture, and food packaging films require a high oxygen (O_2_) and carbon dioxide (CO_2_) barrier to keep the food fresh. However, as shown in Table S1, extensive studies confirmed poor gas and water vapor barrier properties [1,2,3,4,5,6,7,8,9,10,11,12]. In our previous studies, the O_2_, CO_2_ and water vaper permeability coefficients of PBAT45 were reported to be 365 g·mm·m^−2^·day^−1^·atm^−1^, 1.0 barrer and 12.1 barrer, respectively [2,13]. Its water vapor barrier property is only about 1/70 of low density polyethylene (LDPE) [13] and its O_2_ and CO_2_ barrier properties are also far inferior to traditional barrier packaging materials, which are about 1/50 and 1/100 of poly(ethylene terephthalate) (PET) [2].

Poly(butylene succinate-co-terephthalate) (PBST) is another biodegradable aliphatic-aromatic copolyester. In comparison with PBAT, PBST has comparable physical, mechanical properties and biodegradability. But it shows less discoloration during polycondensation and processing and manifests higher glass transition temperature (*T*_g_), which may be beneficial for film blowing and improvement of some properties. In addition, differing from the petroleum-based PBAT, PBST is a partially bio-based polymer because bio-based succinic acid (SA) can be used as a diacid monomer instead of the petroleum-based adipic acid [14]. As the production process of bio-SA consume about 0.37-ton carbon dioxide per ton SA [15], production and applications of PBST is in line with the concept of carbon-neutralization and green and sustainable development. With the rapid process in production technology and continuous reduction in production cost of bio-SA, bio-based PBST will become a promising biodegradable copolyester competing with PBAT in the future. In May 2020, industrial production of PBST was successfully achieved at a scale of 20,000 ton per year in Sinopec Yizheng Chemical Fiber Co. Ltd., China. Modification and application development of PBST are ongoing.

Although PBST has been extensively studied in synthesis, process optimization and reaction kinetics [16,17,18], crystallization and thermal properties [19], mechanical properties, degradability [20] and modification technology [21,22], there is still no report on gas barrier properties of PBST in literature to the best of our knowledge. Considering the potential applications of PBST in agricultural mulch films and packaging materials, investigating and quantitatively evaluating the gas barrier properties of PBST is an essential step for PBST product development. 

In this study, gas permeation coefficients of PBST and PBAT was comparatively studied from theoretical prediction to experimental measurement. Firstly, the O_2_ and CO_2_ permeation coefficients of amorphous and crystalline copolyesters were calculated with group contribution method. Then, the permeation coefficients of O_2_, CO_2_ and water vapor were experimentally measured. It was found that in comparison with PBAT, PBST with close copolymer composition manifested superior barrier properties, especially in O_2_ and CO_2_ barrier. The involved reasons were discussed.

## 2. Prediction of Gas Permeability Coefficients of PBST and PBAT

### 2.1. Theoretical Prediction Method

There are two main models describing the process of gas permeation in materials, namely, the “dissolution-diffusion” model and the “pore flow” model. The former is more commonly used in the gas permeation process in dense non-porous polymer materials. In this model, gas undergoes five steps in the process passing through polymer materials, including surface diffusion, surface adsorption, internal diffusion, surface desorption and surface diffusion [23]. It should be noted that after surface diffusion and adsorption, gas diffusion in the polymer depends on the movement of the polymer chain, and therefore, can only occur in the amorphous region [24]. It can be foreseen that the crystallinity of a semi-crystalline polymer has a significant effect on the gas barrier properties of polymer materials. For the same polymer, the higher the crystallinity, the better the gas barrier performance it has [25,26].

M. Salame correlated polymer gas barrier properties with polymer cohesive energy density, free volume, crystallinity, gas parameters (radius, diffusivity, solubility), etc. [27]. A polymer chain can be divided into many repeat units, and the repeat unit is composed of several groups. For amorphous polymers, the contribution of group i to the overall barrier property is associated with a specific physical quantity “*π*_i_” that characterizes the cohesive energy density and free volume. A comprehensive physical quantity “*π*” of a repeat unit can be calculated from the contribution of all groups (Equation (1)), where *N*_i_ is the number of groups i in the repeat unit. Then, gas permeability coefficient of amorphous polymers (*P*_a_ in unit: cc∙mil∙10^−2^ in^−2^∙day^−1^∙atm^−1^) can be calculated with Equation (2), where *A* and *s* are gas parameters (O_2_, 25 °C: *A* = 8850 cc∙mil∙10^−2^ in^−2^∙day^−1^∙atm^−1^, *s* = 0.112; CO_2_, 25 °C: *A* = 55,100 cc∙mil∙10^−2^ in^−2^∙day^−1^∙atm^−1^, *s* = 0.122.) For a semi-crystalline polymer, as the crystalline region is impermeable for gas, the gas permeability coefficient (*P*) is amended from *P*_a_ with the crystallinity, as shown in Equation (3). Taking 27 common polymers as samples, M. Salame obtained the *π*_i_ values of various groups and thus created the group contribution method to predict gas permeability coefficient of polymer materials using *π*, crystallinity (*x*_c_), gas constants (*A* and *s*) [27]. This method predicts well the permeation behavior of non-polar gases in various polymers [27].

(1) $$\pi =\frac{\sum N_{i}\pi_{i}}{\sum N_{i}}$$

(2) $$P_{a}=Ae^{-s\pi }$$

(3) $$P=P_{a}e^{41.5s\;log(1-x_{c})}$$

In comparison with common gases such as oxygen, nitrogen, air etc., the permeation process of water vapor in polymers is more complicated because water vapor has strong polarity. To date, there is still no universal model to describe or predict permeation of water vapor in polymers though there was some attempt in the literature [28].

As there is still no report on gas permeability coefficients of PBST copolyesters in the literature, the O_2_ and CO_2_ permeability coefficients of PBST was calculated using the group contribution method as the first step of this study. The prediction of PBAT was also undertaken for comparison. The repeat units, groups in the repeat units and their numbers are shown in Table 1. The *π* values of the repeat units (BS, BA, BT) were calculated with Equation (1). For the copolyesters, the *π* values were calibrated with copolymer composition defined as molar percentage of BT repeat unit (*ϕ*_BT_), as shown in Equations (4) and (5).

(4) $$\pi_{\mathrm{PBST}}=\phi_{\mathrm{BT}}\pi_{\mathrm{BT}}+\left(1-\phi_{\mathrm{BT}}\right)\pi_{\mathrm{BS}}$$

(5) $$\pi_{\mathrm{PBAT}}=\phi_{\mathrm{BT}}\pi_{\mathrm{BT}}+\left(1-\phi_{\mathrm{BT}}\right)\pi_{\mathrm{BA}}$$

### 2.2. Prediction of Amorphous Copolyesters

**Figure 1:**
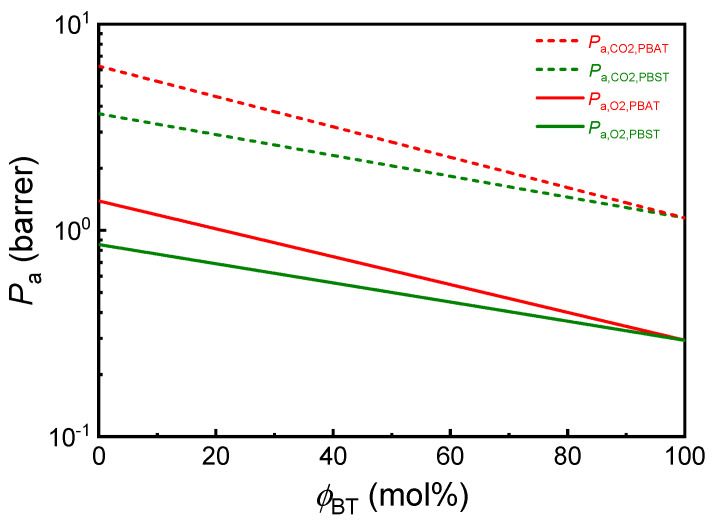
Predicted gas permeability coefficients (*P*_a_, unit: barrer) of amorphous PBST and PBAT copolyesters in full composition range (*ϕ*_BT_ = 0–100 mol%). 1 barrer = 10^−10^ cm^3^∙cm∙cm^−2^∙s^−1^∙cmHg^−1^ = 166.8 cc∙mil∙10^−2^ in^−2^∙day^−1^∙atm^−1^.

Based on the calculated *π* values of respective repeat units, the O_2_ and CO_2_ permeability coefficients (unit: barrer) of completely amorphous PBST and PBAT in full composition range can be directly calculated from copolymer composition with Equations (6)–(9). The results are plotted in Figure 1. The O_2_ and CO_2_ permeability coefficients of PBST45, PBAT45 and corresponding homopolyesters are summarized in Table S2. As the *π* value of BS, BA and BT repeat units (*π*_BA_ = 32.4, *π*_BS_ = 36.8, *π*_BT_ = 46.3.) increases in order, the O_2_ and CO_2_ permeability coefficients of the homopolyesters obey the reverse order: PBT < PBS < PBA. In comparison with amorphous PBA, amorphous PBS and PBT have 1.6 and 4.8 times O_2_ barrier and 1.7- and 5.4-times CO_2_ barrier properties, respectively. Therefore, in the amorphous copolyesters, BS unit has greater contribution than BA unit and BT unit contributes the most to the gas barrier of the materials. Consequently, the *P*_a_ values of both copolyesters decrease with the increase of BT molar fraction (*ϕ*_BT_) and the *P*_a_ values of amorphous PBST are always lower than those of amorphous PBAT at the same copolymer composition. At a typical composition of *ϕ*_BT_ = 45 mol% equal or close to that of commercial products, Ecoflex^®^, the *P*_a,O_2__ and *P*_a,CO_2__ values of amorphous PBAT45 are 0.69 and 2.92 barrer respectively, and those of amorphous PBST45 are 0.52 and 2.18 barrer respectively. The O_2_ and CO_2_ barrier properties of amorphous PBST45 are 1.33 and 1.34 times of amorphous PBAT45, respectively.

(6) $$logP_{O_{2},\mathrm{PBST}}=-0.462\phi_{\mathrm{BT}}-0.065$$

(7) $$logP_{{\mathrm{CO}}_{2},\mathrm{PBST}}=-0.505\phi_{\mathrm{BT}}+0.566$$

(8) $$logP_{O_{2},\mathrm{PBAT}}=-0.681\phi_{\mathrm{BT}}+0.143$$

(9) $$logP_{{\mathrm{CO}}_{2},\mathrm{PBAT}}=-0.857\phi_{\mathrm{BT}}+0.797$$

### 2.3. Prediction of Crystalline Copolyesters

Taking crystallinity (*x*_c_) into account, O_2_ and CO_2_ permeability coefficients (*P*_O_2__, *P*_CO_2__) of PBST and PBAT copolyesters in full range of compositions and crystallinity were also calculated. The results are shown in Figure S1. In theory, the *p* values of PBST and PBAT all decrease significantly with the increase of crystallinity, especially at high crystallinity range. However, in fact, both biodegradable PBAT and PBST with practical thermal and mechanical properties are weakly crystallizable copolyesters with low crystallinity, often less than 20%. When PBST45 and PBAT45 with 20% crystallinity are concerned, they show 1.56 and 1.64 times O_2_ and CO_2_ barrier to the amorphous counterparts. 

**Figure 2:**
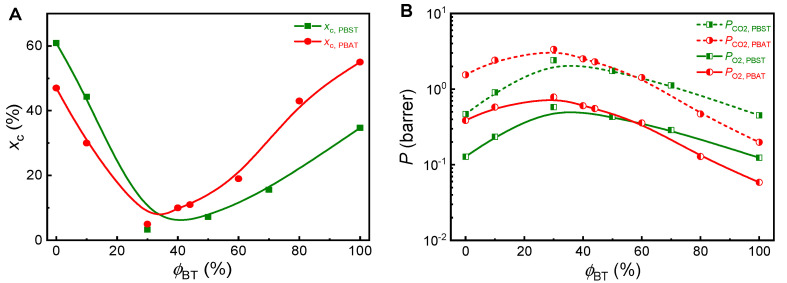
(**A**) Crystallinity (*x*_c_) of PBAT and PBST copolyesters changes with composition (*ϕ*_BT_ = 0–100 mol%, data from refs. [19,29]) and (**B**) the O_2_ and CO_2_ permeability coefficients (*P*_O_2__, *P*_CO_2__) of the PBAT and PBST copolyesters predicted from the crystallinity data.

To predict the gas permeability coefficients of practical PBST and PBAT products, the true crystallinity must be measured and considered. It is well known that the composition has a great influence on the crystallinity of PBST and PBAT. With the increase of *ϕ*_BT_, the crystallinity of these copolymers decreases first and then increases again, showing a minimum value at *ϕ*_BT_ of 30–40%. When *ϕ*_BT_ is less than 20%, only BA or BS segments exist in crystals of copolymers; when *ϕ*_BT_ is 20–30%, BT segment begins to appear in the crystal; when *ϕ*_BT_ is higher than 30–40%, only BT segment exists in the crystals [19,29]. The crystallinity data from some literature [19,29] are shown in Figure 2A and used for predicting gas permeability coefficients.

Based on the real crystallinity data of PBST and PBAT at different compositions, the gas permeability coefficients of PBST and PBAT are calculated and shown in Figure 2B. Both copolyesters show maximum gas permeability coefficients, or in other words, minimum gas barrier at *ϕ*_BT_ of 30–40%. These results reflect the comprehensive influence of composition and crystallinity on barrier properties. When *ϕ*_BT_ is less than 30–40%, the increase in *ϕ*_BT_ has a positive effect on gas barrier, but the decreasing crystallinity has a negative impact on gas barrier. In comparison, the negative effect of crystallinity is predominant and as a result, the gas barrier properties decrease with *ϕ*_BT_. When *ϕ*_BT_ is greater than 30–40%, the increase in both *ϕ*_BT_ and crystallinity have synergistic positive effect on the barrier properties, therefore, the gas permeability coefficient decreases with *ϕ*_BT_. Besides, it can be seen that PBST has higher gas barrier than PBAT at *ϕ*_BT_ < 60%, but PBAT shows higher gas barrier than PBST at *ϕ*_BT_ > 60%, due to its clearly higher crystallinity at *ϕ*_BT_ > 60%.

From the above prediction results, it can be concluded that amorphous PBST has slightly higher gas barrier properties than amorphous PBAT at the same copolymer composition, but practical semi-crystalline PBST may has slightly higher or lower gas barrier when compared with semi-crystalline PBAT, depending on the crystallinity.

## 3. Experimental

### Materials

Poly(butylene adipate-co-butylene terephthalate) (PBAT48) and poly(butylene succinate-co-terephthalate)s (PBST44 and PBST61) were provided by Sinopec Yizheng Chem Fiber. Co. (SYCF), Yizheng, China. PBST23, PBST33 and PBST71 were synthesized in our lab via a coesterification-copolycondensation process from 1,4-succinic acid (SA, 99.9%, Anhui Sanxin Chem. Co., Chizhou, China), terephthalic acid (TPA, 99.9%, Hengyi PetroChem. Co., Hangzhou, China) and 1,4-butanediol (BDO, 99.9%, SYCF. Co., Yizheng, China) using tetrabutoxyl titanium (TBT, 99.8%, J&K Chemical, Shanghai, China) as catalyst. The detailed process was reported previously [16,21,22]. The intrinsic viscosity and copolymer composition of the copolyesters are listed in Table 2. Anhydrous calcium chloride (CaCl_2_, 99%, Ruichen Chemical, Jinan, China) and chloroform (AR grade, Sinopharm Chem. Reagent Co., Shanghai, China) were all used as received.

## 4. Characterization

Intrinsic viscosity of the copolyesters was measured at 25 °C using IVS300 semi-automatic viscometer tester (Hangzhou Zhongwang Co., Hangzhou, China) equipped with an Ubbelohde viscometer (inner diameter 0.36 mm), with 0.125 g polymer dissolved in 25 mL chloroform as a solution sample. ^1^H NMR spectra were recorded with a Bruker AC-80 spectroscopy (400 M, Karlsruhe, Germany). Deuterated chloroform was used as solvent and tetramethylsilane as internal reference. The copolymer composition of the copolyesters were calculated from the ^1^H NMR results (Figure S2) according to previously reported method [19,20,21,22]. 

Polymer films with thickness of about 300–400 μm were prepared by thermal pressing at 150–200 °C and used for crystallinity and gas permeation measurements. Thermal transition behaviors were recorded with differential scanning calorimetry (DSC, Q200, TA Instrument, Newcastle, America) under nitrogen flow. Approximately 6–8 mg of film sample was scanned with a heating rate of 10 °C/min. The melting enthalpy from the first heating curve (Δ*H*_m_) was obtained and used to calculate the crystallinity, *x*_c_ = Δ*H*_m_/Δ*H*_m_^0^, in which Δ*H*_m_^0^ is the melting enthalpy of 100% crystalline polymer cited from ref. [4,19]. The rigid amorphous fraction (RAF) *x*_RAF_ was calculated from crystallinity *x*_c_ and amorphous fraction *x*_a_, namely, *x*_RAF_ = 1 − *x*_c_ − *x*_a_. The amorphous fraction *x*_a_ (=Δ*C*_p_/Δ*C*_p−a_) was calculated from the specific heat capacity differences after and before glass transition of semi-crystalline (did not erase heat history) sample (Δ*C*_p_) and completely amorphous sample obtained by rapid cooling (Δ*C*_p−a_).

O_2_ and CO_2_ transmission rates (*GTR*) were measured with differential pressure method at 23 °C after 8 h vacuuming of test chambers and film samples, using a BSG-33E gas permeability tester (Labstone Instruments Technology Co. Guangzhou, China). The gas permeability coefficients (*P* = *GTR*.*d*/Δ*P*) were calculated from *GTR*, film thickness (*d*) and pressure difference (Δ*P*) between both sides of the film. The diffusion coefficient (*D*) and solubility (*S*) of O_2_ and CO_2_ were also obtained during *P* measurement from *D* = *d*^2^/(6*θ*) and *S* = *P*/*D*, where *d* is film thickness, *θ* is time lag calculated from pressure curve. Water vapor transmission rate (*WVTR*) was measured with dish method following ASTM E96-16. A metal cup filled with dried CaCl_2_ as desiccant was sealed with a sample film. The testing cup was put in a chamber controlled at constant temperature (38 °C) and humidity (90 RH%). Water vapor permeated gradually from the chamber into the cup through the film and was rapidly adsorbed by CaCl_2_. Total weight of the testing cup was recorded over time to calculate *WVTR*. Water vapor permeability (*P*_WV_ = *WVTR*.*d*/[*P*^0^(*RH*_1_-*RH*_2_)]) was calculated from *WVTR*, film thickness (*d*), saturated water vapor pressure (*P*^0^ = 6630 Pa at 38 °C) and relative humidity at both sides of the film (*RH*_1_ = 90%, *RH*_2_ = 0%). All barrier test results are from the average of three parallel experiments.

Positron annihilation lifetime spectroscopy (PALS) experiment was performed with PLS-System 414A (Beijing Zhongjian Weikang Technology Co., Ltd., Beijing, China ), using a film sample with size of 1.5 × 1.5 × 0.1 cm^3^ made by thermal-pressing. Positron source was sandwiched between two identical specimens. The data were recorded with total counts of 2.5 million within the test time of 20 ns and analyzed by standard LT9.0 and MELT4.0 programs.

Small angle X-ray scattering experiment was performed with Xeuss SAXS/WAXS system (XENOCS, Grenoble, France) at test power of 50 KV and 0.6 mA, using a film sample with size of 1 × 1 × 0.1 cm^3^ made by thermal-pressing. The test time was 1200 s, the detection wavelength was 0.15148 nm and detector-sample distance was 1370 mm. The test based on a point light source and PILATUS 100 K pixel detector was used to record the scattered signal.

Dynamic mechanical behavior was analyzed with a TA Q800 instrument (TA Instrument, Newcastle, America) at work frequency of 1 Hz, using a film sample with size of 2.5 × 1 × 0.015 cm^3^ made by thermal-pressing. After a rapid cooling, the dynamic spectrum was detected by heating the sample at 4 °C/min in the temperature range from −100 °C to 60 °C. The tan*δ* was recorded as a function of temperature. 

Water contact angle of the copolyesters was measured with an OCA 20 video optical contact angle measuring device (Dataphysics Co., Filderstadt, Germany). Deionized water (0.36 μL) was dropped at three locations on a film with flat surface placed on the sample table to measure the water contact angle.

## 5. Results

**Figure 3:**
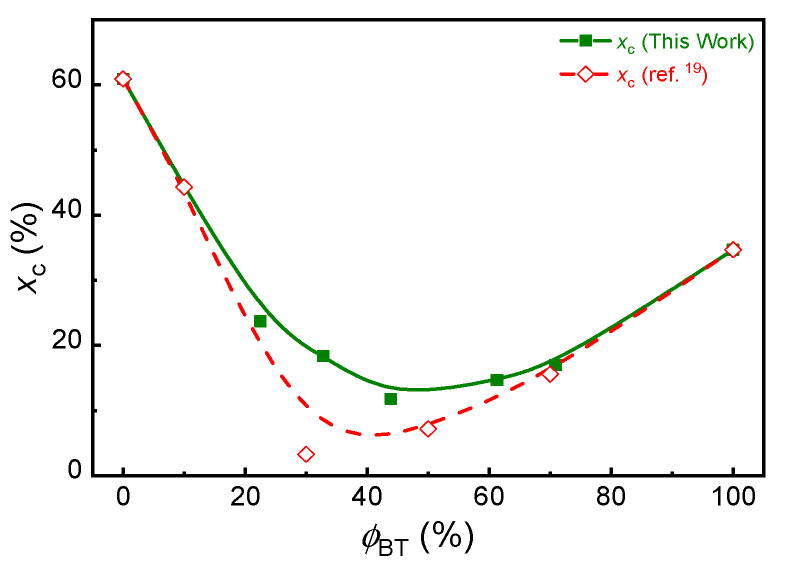
Crystallinity (*x*_c_) of different PBST (*ϕ*_BT_ = 0–100 mol%) films (the data of open points from ref. [19]).

In some previous studies of PBST and PBAT, it has been found that the melting point (*T*_m_) and enthalpy (Δ*H*_m_) of the PBST copolyesters initially decreased with an increase of *ϕ*_BT_, and then tended to increase near to the PBT values when the BT unit became the dominant component [19,29]. In this study, considering the effect of crystallinity on gas barrier, the crystallinity of some PBST film samples ready for barrier experiment was measured with DSC. The DCS first heating curves are shown in Figure S3, and the calculated crystallinity data are summarized in Table S3. Similar composition dependence of crystallinity was observed in Figure 3 when compared with the literature results [19], but the crystallinity of these film samples at middle composition is clearly higher than that in previous report [19]. Obviously, this was related to the heat history during the thermal-pressing process.

**Figure 4:**
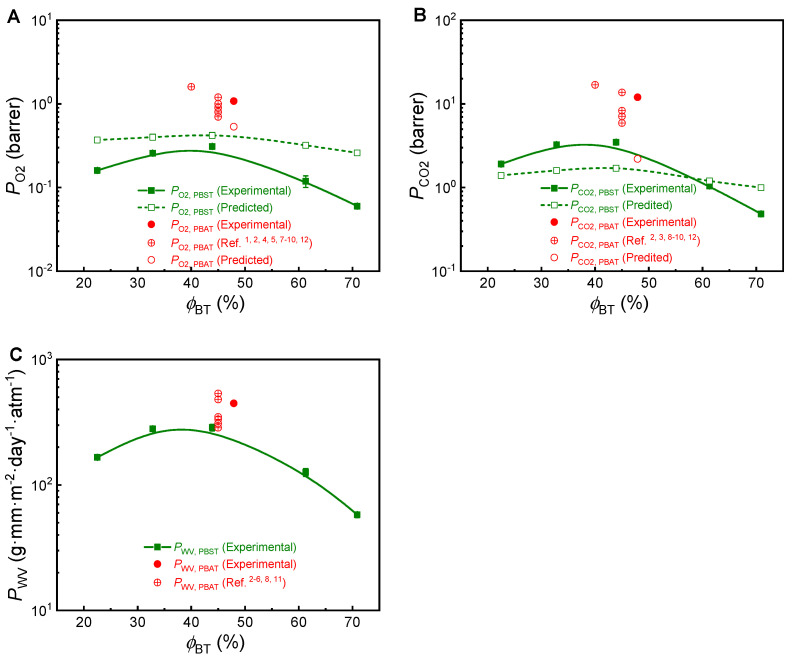
Permeation coefficients of (**A**) oxygen (*P*_O_2__), (**B**) carbon dioxide (*P*_CO_2__) and (**C**) water vapor (*P*_WV_) in PBST and PBAT. Table 3. and the literature data are from refs. [1,2,3,4,5,6,7,8,9,10,11,12] and also summarized in Table S1.

Using the crystallinity data, the O_2_ and CO_2_ permeation coefficients (*P*_O_2__, *P*_CO_2__) were calculated with the group contribution method. The results are shown in Figure 4. The experimental results are summarized in Table 3 and also plotted with copolymer composition, as shown in Figure 4.

All the calculated and experimental permeation coefficients of PBST increase first with increasing *ϕ*_BT_, reach a maximum and then decrease faster with further increasing *ϕ*_BT_. Obviously, such a trend is ascribed to the joint contribution of copolymer composition and the crystallinity as explained above. When the composition dependence of calculated permeation coefficient of PBST is concerned, the results in Figure 4 are clearly weaker than those in Figure 2. In other words, the calculated permeation coefficient of PBST in Figure 4 changes weaker in the same composition range. Clearly, such difference is ascribed to the smaller change of crystallinity of PBST with copolymer composition in this study, as shown in Figure 3.

The experimental values of *P*_O_2__, *P*_CO_2__ and *P*_WV_ of these PBSTs containing 23–71 mol% BT range 0.06–0.31 barrer, 0.48–3.48 barrer and 58–287 g·mm·m^−2^·day^−1^·atm^−1^, respectively. It can be seen that the experimental values of *P*_O_2__ are clearly lower than the calculated values in the experimental composition range. In other words, the oxygen barrier performance of PBST is unexpectedly superior to that predicted from group contribution method. Differently, the experimental value of *P*_CO_2__ is higher than the calculated values at *ϕ*_BT_ < 57 mol% but becomes lower than the calculated values at *ϕ*_BT_ > 57 mol%.

PBAT copolyesters with *ϕ*_BT_ of 45–48 mol% has been commercially available for over two decades and widely used as biodegradable polymers nowadays. They are well-known for poor gas and water vapor barrier for packing and mulch film applications. The *P*_O_2__, *P*_CO_2__ and *P*_WV_ values of PBAT copolyesters reported in the literature range 0.7–1.6 barrer, 5.9–16.9 barrer, and 285–536 g·mm·m^−2^·day^−1^·atm^−1^, respectively (Table S1). The experiment results of a PBAT48 sample in this study, *P*_O_2__ of 1.08 barrer, *P*_CO_2__ of 12.1 barrer and *P*_WV_ of 447 g·mm·m^−2^·day^−1^·atm^−1^, fall within the range reported in the literature. As shown in Table 4, comparing the experimental results of PBAT48 and PBST44 which have similar copolymer composition, it can be seen that the *P*_O_2__, *P*_CO_2__ and *P*_WV_ values of PBST44 are 1/3.48, 1/3.48 and 1/1.56 of those of PBAT48. In other words, the PBST44 copolyester manifested O_2_, CO_2_ and water vapor barrier properties 3.48, 3.48 and 1.56 times to PBAT48. Obviously, the gas and water vapor barrier performance of PBST is clearly superior to PBAT. The improvement degree (3.48 times) in O_2_, CO_2_ barrier is higher than the predicted result (1.30 times).

As far as PBST44 and PBAT48, the two copolyesters with the same BT units’ volume percent (38 vol%), are concerned, such big difference in gas barrier could not be ascribed to the small difference in crystallinity (11.8% vs. 10.0%) and *ϕ*_BT_ (44% vs. 48%) between them. In fact, it can be calculated from the group’s contribution method that the *P*_O_2__ will increase by 6% when the *ϕ*_BT_ of PBAT changes from 48% to 44%, and the *P*_O_2__ of PBAT48 will rise only by 2% when its crystallinity changes from 10.0% to 11.8%. Therefore, the slight differences in composition and crystallinity of PBST44 and PBAT48 have negligible influence on their barrier properties. To make clear the factor determining the big difference in gas barrier, more characterizations and analyses were conducted and the results are discussed as follows.

## 6. Discussion

### 6.1. Segment Movement and Gas Diffusion

**Figure 5:**
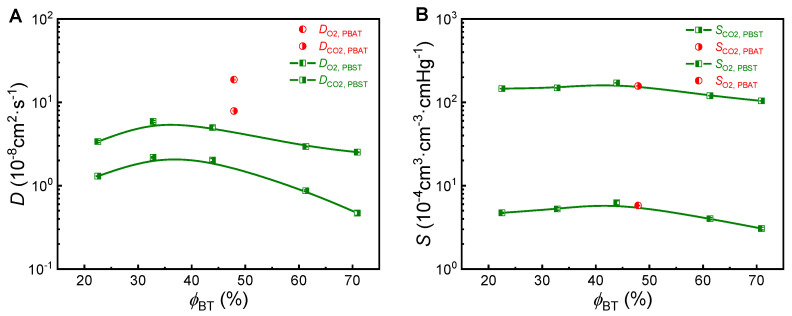
(**A**) Diffusion coefficient (*D*_O_2__, *D*_CO_2__) and (**B**) Solubility (*S*_O_2__, *S*_CO_2__) of PBST and PBAT.

According to the dissolution-diffusion theory, a permeation coefficient is equal to the product of corresponding gas diffusion coefficient and solubility. To make clear which is the key factor for the superior gas barrier of PBST, the diffusion coefficients (*D*_O_2__, *D*_CO_2__) and solubility (*S*_O_2__, *S*_CO_2__) data were also obtained from the permeation experiments of O_2_ and CO_2_ and plotted with copolymer composition in Figure 5. It can be seen that they both show composition dependence similar to that of permeation coefficients. Therefore, the composition dependence of the permeation coefficients is the joint contribution of diffusion coefficients and solubility. On the other hand, it can be seen that PBST44 and PBAT48 have almost the same gas solubility but very different diffusion coefficient. The O_2_ and CO_2_ diffusion coefficients of PBST44 are much smaller than those of PBAT48. In other words, gas diffusion in PBST44 is much slower than in PBAT48. This result implies that the chain segment movement in PBST44 is much slower than in PBAT48.

**Figure 6:**
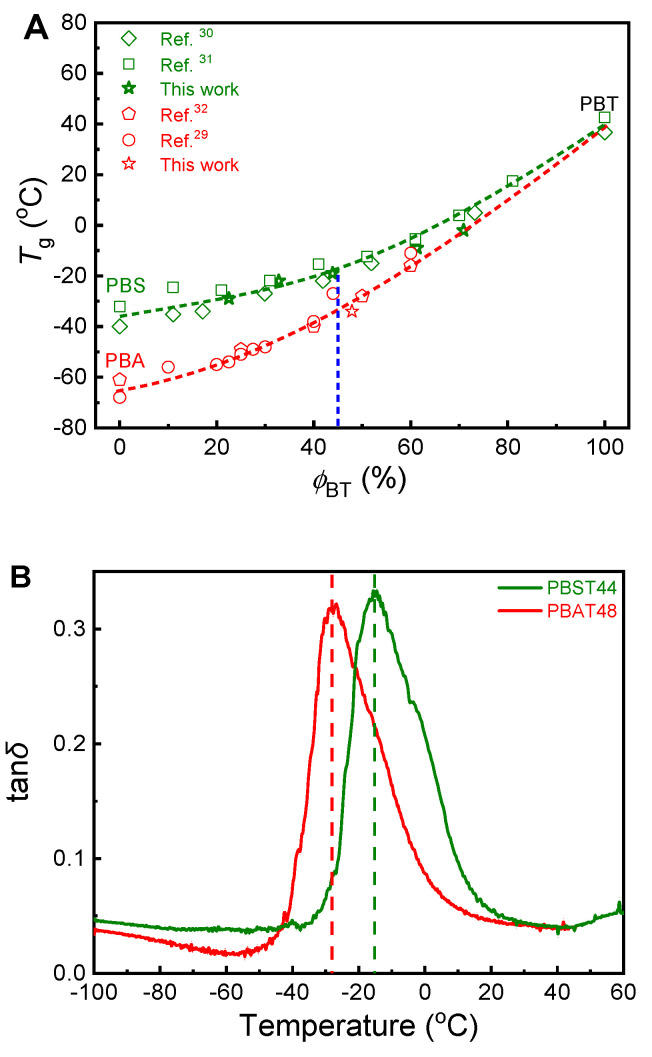
(**A**) Composition dependence of glass transition temperature (measured from DSC) of PBST and PBAT copolyesters, data from refs. [29,30,31,32]; (**B**) tan*δ* of PBAT48 and PBST44 in temperature range of −100 °C to 60 °C.

The only difference in the chemical structure of PBST and PBAT is the number of CH_2_ groups in the diacid structural units: 2 for PBST and 4 for PBAT. The shorter CH_2_ groups in PBST results in slower segment movement and therefore higher glass transition temperature (*T*_g_) than PBAT. It is well known that PBST has higher *T*_g_ than PBAT at the same copolymer composition. Figure 6A summarizes the composition dependence of PBST and PBAT *T*_g_ data measured from DSC in this study and from literature. The *T*_g_ of both kinds of copolyesters increases with increasing *ϕ*_BT_. Particularly, PBST44 has a *T*_g_ about 15 °C higher than PBAT48.

It is well-known that the barrier properties of polymers are also affected by free volume, which mainly affects segment movement and gas diffusion and is influenced by *T*_g_ and crystallinity [33,34]. The free volume in polymers refers to the part of the volume that is not occupied by polymer chains, and it is the prerequisite for chains movement to drive gas diffusion. According to the theory of J. L. Duda and J. M. Zielinski, free volume includes interstitial free volume and hole free volume 34. The free volume of polymers increases with the increase of temperature, especially when the temperature is above *T*_g_; the increase rate of hole free volume is more significant above *T*_g_ [34]. As mentioned above, PBST44 and PBAT48 have similar crystallinity, while PBST44 has a *T*_g_ about 15 °C higher than PBAT48, and both *T*_g_ values are lower than room temperature. Therefore, at the application or test temperature, PBST44 inevitably has less free volume than PBAT48.

This result can be confirmed by the positron annihilation lifetime spectroscopy (PALS), a powerful tool for analyzing the free volume of polymer materials. After injecting into materials, positrons can exist in two forms: free positron and positronium (Ps). Ps can be divided into parapositronium (p-Ps) and orthopositronium (o-Ps) [33]. The annihilation lifetimes (*τ*_1_, *τ*_2_) of p-Ps are generally not sensitive to the internal structure of the materials, but the annihilation lifetime of o-Ps (*τ*_3_) is positively correlated with the pore size inside the materials [33]. The intensities (*I*_1_*, I*_2_, *I*_3_) of Ps are reflected by the counts; among them, the intensity of o-Ps (*I*_3_) is positively correlated with the total free volume of the materials 33. The PALS of PBAT48 and PBST44 were tested and shown in Figure S4, the intensity and annihilation lifetime of o-Ps (*τ*_3_, *I*_3_) were analyzed and listed in Table 5. The radius of free volume pore (*R*), volume of single pore (*V*_F_), total free volume fraction (*f*) calculated according to Equations (10–12), respectively, are also summarized in Table 5. The electron layer thickness (Δ*R*) and arbitrary scaling factor (*C*) for spherical cavity were taken from the Ref. [33], being 0.166 nm and 1.5 nm^−3^, respectively.

(10) $$\tau_{3}=\frac{1}{2}{\left[1-\frac{R}{R+\Delta R}+\left(\frac{1}{2\pi }\right)sin\left(\frac{2\pi R}{R+\Delta R}\right)\right]}^{-1}$$

(11) $$V_{F}=\frac{4\pi R^{3}}{3}$$

(12) $$f=CV_{F}I_{3}$$

Clearly, PBST44 has smaller single pore free volume (0.09 nm^3^ vs. 0.11 nm^3^) and smaller total free volume fraction (1.38% vs. 1.76%) than PBAT48. Usually, the free volume of polymers accounts for only a small proportion (1–10%) but has a huge influence on segment movement [33]. In this study, although both the free volume fractions are relatively small, there are still obvious differences in comparison. Obviously, the lower free volume fraction of PBST accounts for the smaller diffusion coefficient and gas permeability coefficient shown in Table 3 and discussed above.

### 6.2. β-Transition

Besides the chain segment movement, *β*-transition resulted from stretching vibration of bond length, deformation vibration of bond angle and twisted vibration of C–C single bond may also affect gas diffusion in polymers [26]. For this reason, a dynamic mechanical analysis of PBST44 and PBAT48 was conducted in the temperature range from −100 °C to 60 °C. The results shown in Figure 6B also indicate higher glass transition temperature of PBST (−16 °C for PBST44 vs. −28 °C for PBAT48) and therefore slower segment movement of PBST. Unfortunately, *ꞵ*-relaxation transition was not observed in low temperature zone up to −100 °C for both copolyesters. In fact, *β*-relaxation transition of PBST and PBAT has also not been reported in literature [20,35]. The *β*-relaxation of PBST44 and PBAT48 may appear in the temperature range below −100 °C which is out of the detection temperature range of the DMA instrument, and therefore its influence still cannot be completely excluded.

### 6.3. Rigid Amorphous Phase Fraction

For a semi-crystalline polymer, there often exists a rigid amorphous phase between amorphous phase and crystalline phase. Rigid amorphous phase is also regarded to have a barrier effect similar to that of the crystalline phase [33,36,37]. It is a simple way to get the rigid amorphous phase fraction (RAF) from the Δ*C*_p_ observed in the DSC. The rigid amorphous fraction *x*_RAF_ was calculated from crystallinity *x*_c_ and amorphous fraction *x*_a_, namely, *x*_RAF_ = 1 − *x*_c_ − *x*_a_. The amorphous fraction *x*_a_ (=Δ*C*_p_/Δ*C*_p−a_) was calculated from the specific heat capacity differences after and before glass transition of the semi-crystalline (did not erase heat history) sample (Δ*C*_p_) and completely amorphous sample obtained by rapid cooling (Δ*C*_p−a_) [37]. For PBST44, the RAF result (33%) was obtained from the heat capacity differences (Δ*C*_p_, Δ*C*_p−a_) of semi-crystalline and completely amorphous samples. But for PBAT48, unfortunately, due to very rapid crystallization (though crystallinity is not high), it was unsuccessful obtaining amorphous samples, even under rapid cooling, which is the prerequisite for testing RAF via DSC. Therefore, it is impossible to compare the RAF data of both copolyesters from DSC. The DSC scan and heat capacity vs. temperature curves was provided in Figure S5.

As small angle X-ray scattering (SAX) is a powerful tool for characterizing the microphase structure of a polymer, SAX patterns of PBST44 and PBAT48 were recorded and the crystalline-amorphous-rigid structure was analyzed in terms of one-dimensional stack model and one-dimensional correlation function [38] to explore if there is clear difference in rigid amorphous phase fraction (RAF) between them.

**Figure 7:**
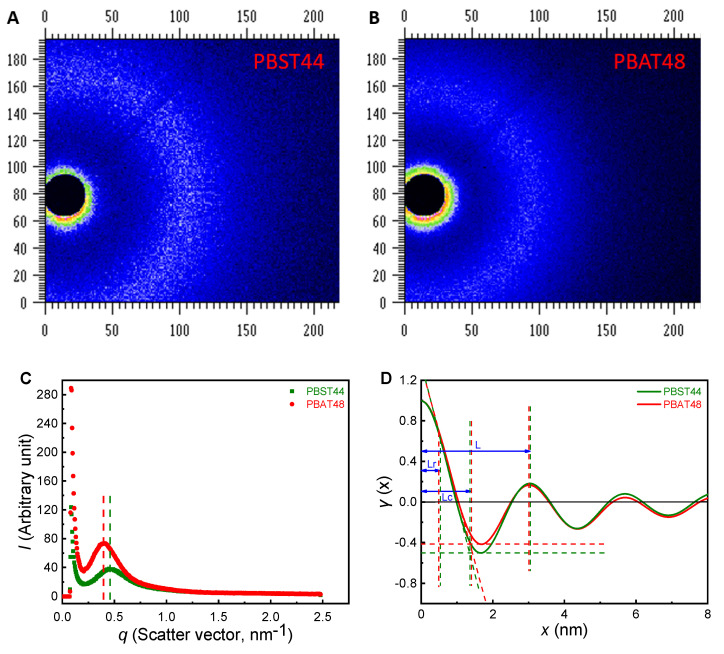
Two-dimensional SAXs patterns of (**A**) PBST44 and (**B**) PBAT48, (**C**) one-dimensional exported data and (**D**) one-dimensional pattern.

The two-dimensional patterns of PBAT48 and PBST44 are shown as Figure 7A,B, the correlation relationships of “scattering intensity-scattering vector” derived from them and the one-dimensional models are shown in Figure 7C,D, respectively. It can be seen that, except for the peak scattering intensity, the SAXs patterns of PBST44 and PBAT48 are basically the same. After fitting, denoising and subtracting the background [38], a modified one-dimensional pattern is obtained via Equations (13) and (14) as shown in Figure 7D. Based on the “characteristic triangle” of the pattern [39], three-phase (crystalline, amorphous, rigid amorphous) data were obtained. The results are shown in Table 6. It can be seen that PBST44 and PBAT48 have close three-phase structure and rigid amorphous phase fraction (17.7% vs. 16.3%). Therefore, it can be deduced that the tiny difference in the aggregated structure between them are not the main cause of the large difference in the O_2_ and CO_2_ barrier properties of PBST44 and PBAT48.

(13) $$Q=\int_{0}^{\infty }Iq^{2}dq$$

(14) $$\gamma \left(x\right)=\frac{1}{Q}\int_{0}^{\infty }Iq^{2}\cos(qx)dq$$

### 6.4. Effect of Hydrophilicity on WVP

**Figure 8:**
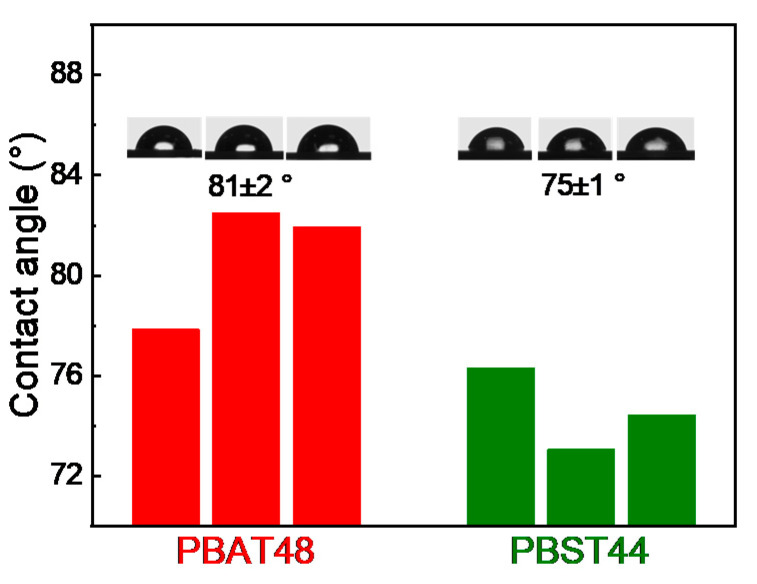
Static hydrophilic contact angle of PBAT48 and PBST44.

Although the O_2_ and CO_2_ barrier properties of PBST44 are 3.5-times to that of PBAT48, the water vapor barrier property of PBST44 is only 1.5-times. The lesser improvement in the water vapor barrier may result from the differences in the hydrophilicity of PBST and PBAT, as there are two less hydrophobic methylene groups in PBST. To make it clear, a static hydrophilic contact angle test was conducted. The results shown in Figure 8 indicate that PBST44 has a smaller hydrophilic contact angle than PBAT48 (75 ± 1° vs. 81 ± 2°), suggesting its stronger hydrophilicity and therefore easier dissolution of water vapor in it. The increase of diffusion resistance caused by the more rigid chain segment and the increase in solubility caused by the stronger hydrophilicity of PBST cancel out each other to some degree, consequently, PBST shows a smaller improvement in the water vapor barrier than in the O_2_ and CO_2_ barrier.

## 7. Conclusions

Oxygen, carbon dioxide and water vapor permeation coefficients (*P*_O_2__, *P*_CO_2__ and *P*_WV_) of PBST with 23–71 mol% BT unit and PBAT with 48 mol% BT unit were comparatively studied, and the *P*_O_2__, *P*_CO_2__ values were also theoretically predicted with group contribution method considering both contribution of groups and crystallinity. *P*_O_2__, *P*_CO_2__ and *P*_WV_ of PBST all show clear copolymer composition (*ϕ*_BT_) dependence, first increasing and then decreasing with *ϕ*_BT_, reaching maximum values at *ϕ*_BT_ of 30–40 mol% when the BT unit became the dominant unit for crystallization. The bigger contribution of aromatic group than aliphatic groups to gas barrier and the nonlinear composition dependence of crystallinity account for the composition dependence of permeation coefficients. The predicted *P*_C__O_2__ values of PBSTs agree with the measured ones, but the predicted *P*_O_2__ values of PBSTs are clearly higher than the measured *P*_O_2__. When compared with PBAT48, PBST44 with close composition and crystallinity shows 3.5-times O_2_ and CO_2_ and 1.5-times water vapor barrier properties. For the superior O_2_ and CO_2_ barrier, the slower chain segment movement and less free volume, and therefore slower gas diffusion in PBST is believed to be the main reason. It results from two less methylene groups in succinate unit in PBST and is supported by higher glass transition. Possible effect of rigid amorphous phase fraction was excluded as there is no clear difference in rigid amorphous phase fraction observed by SAX between of the two copolyesters. Though there is no *β*-transition detected by DMA in accessible temperature range, it is believed that the *β*-relaxation of PBST44 and PBAT48 may appear in the temperature range below −100 °C, and its influence cannot be completely excluded. On the other hand, the two less methylene groups in succinate unit in PBST lead to stronger hydrophilicity, which strengthens the water vapor dissolution and partially offsets the weakening of diffusion. As a result, the improvement in the water vapor barrier in PBST is not as high as the improvement in the O_2_ and CO_2_ barrier.

## Figures and Tables

**Table 1:** Repeat units, group numbers in repeat units and physical quantity “*π*” of repeat units of PBST and PBAT copolyesters.

Copolyester	Repeat Unit	Group Numbers in Repeat Unit, *N*_i_^a^			*π* ^b^
		CH_2_	COO	Phenylene	
PBST	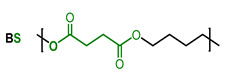	6	2	0	36.8
	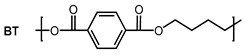	4	2	1	46.3
PBAT	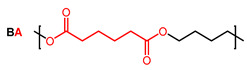	8	2	0	32.4
	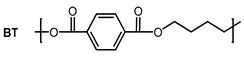	4	2	1	46.3

^a^: “*N*_i_” is the number of groups “i” in the repeating unit. ^b^: *π* value of BS, BA and BT repeat units calculated with Equation (1), in which the *π*_i_ value of CH_2_, COO and phenylene groups are *π*_1_ = 15, *π*_2_ = 102 and *π*_3_ = 60, respectively, cited from ref. [27].

**Table 2:** Intrinsic viscosity (*IV*) and copolymer composition (*ϕ*_BT_) of the copolyesters.

Sample	*IV*^a^ (dL/g)	*ϕ*_BT_^b^ (mol%)	Source
PBST23	1.15	22.5	This lab ^c^
PBST33	1.17	32.8	This lab ^c^
PBST44	0.69	43.9	SYCF
PBST61	0.57	61.3	SYCF
PBST71	0.77	70.9	This lab ^c^
PBAT48	0.65	47.9	SYCF

^a^: Measured in chloroform as solvent at 25 °C. ^b^: Copolymer composition expressed by molar percentage of BT repeat unit. ^c^: calculated from ^1^H NMR spectra which are showed in Figure S2.

**Table 3:** Measured gas permeation coefficients (*P*_WV_, *P*_O_2__, *P*_CO_2__), diffusion coefficients (*D*_O_2__, *D*_C__O_2__) and solubility (*S*_O_2__, *S*_CO_2__) of PBST and PBAT48 copolyesters.

Sample	*P*_WV_ ^a^	*P*_O_2__ ^b^	*D*_O_2__ ^c^	*S*_O_2__ ^d1^	*P*_CO_2__ ^b^	*D*_C__O_2__ ^c^	*S*_C__O_2__ ^d2^
PBST23	166 ± 1	0.160 ± 0.007	3.38 ± 0.10	4.72 ± 0.07	1.91 ± 0.031	1.30 ± 0.01	1.46 ± 0.03
PBST33	280 ± 2	0.257 ± 0.000	5.89 ± 0.17	5.26 ± 0.18	3.26 ± 0.074	2.19 ± 0.04	1.49 ± 0.05
PBST44	287 ± 17	0.310 ± 0.010	4.98 ± 0.22	6.24 ± 0.35	3.48 ± 0.239	2.03 ± 0.09	1.71 ± 0.02
PBST61	126 ± 9	0.119 ± 0.004	2.95 ± 0.02	4.02 ± 0.11	1.05 ± 0.017	0.87 ± 0.02	1.20 ± 0.01
PBST71	58 ± 3	0.060 ± 0.001	2.52 ± 0.02	3.06 ± 0.11	0.48 ± 0.009	0.47 ± 0.03	1.04 ± 0.01
PBAT48	447 ± 12	1.079 ± 0.010	18.7 ± 0.26	5.78 ± 0.03	12.1 ± 0.010	7.86 ± 0.02	1.57 ± 0.03

^a^: g·mm/(m^2^·day·atm), measured at 38 °C, 90 RH%. ^b^: barrer, 1 barrer = 10^−10^ cm^3^·cm/(cm^2^·s·cmHg), measured at 23 °C, 0 RH%. ^c^: 10^−^^8^ cm^2^·s^−^^1^. ^d1^: 10^−^^4^ cm^3^·cm^−^^3^·cmHg^−^^1^. ^d2^: 10^−^^2^ cm^3^·cm^−^^3^·cmHg^−^^1^.

**Table 4:** Barrier improvement factors of PBST44 and PBAT48 copolyester.

Sample	*BIF*_O_2_,exp_ ^a^	*BIF*_O_2_,cal_ ^a^	*BIF*_CO_2_,exp_ ^a^	*BIF*_CO_2_,cal_ ^a^	*BIF*_WV,exp_ ^a^
PBAT48	1	1	1	1	1
PBST44	3.48	1.30	3.48	1.30	1.56

^a^: Barrier improvement factor.

**Table 5:** Positron annihilation lifetime spectroscopy (PALS) results related to free volume of PBAT48 and PBST44.

Sample	*I*_3_ ^a^ (%)	*τ*_3_ ^b^ (ns)	*R* ^c^ (nm)	*V*_F_ ^d^ (nm^3^)	*f*^e^ (%)
PBAT48	10.79	2.13	0.30	0.11	1.76
PBST44	9.96	1.95	0.28	0.09	1.38

^a^: The intensity of o-Ps. ^b^: The annihilation lifetime of o-Ps. ^c^: The radius of free volume pore. ^d^: The volume of single pore. ^e^: The total free volume fraction.

**Table 6:** Three-phase layer thickness of PBAT48 and PBST44 calculated from SAXS results.

Sample	*L*^a^ (nm)	*L*_C_^b^ (nm)	*L*_R_ ^c^ (nm)	*L*_A_ ^d^ (nm)
PBAT48	3.00	1.40	0.49	1.11
PBST44	3.05	1.36	0.54	1.15

^a^: thickness of a long cycle. ^b^: thickness of a crystalline layer. ^c^: thickness of a rigid amorphous phase layer. ^d^: thickness of an amorphous phase layer.

## Data Availability

Exclude this statement.

## Supplementary Materials

The following are available online at https://www.mdpi.com/article/10.3390/polym13193449/s1, Table S1: PBAT gas barrier data reported in literature, Table S2: Gas permeability coefficients of some amorphous homopolyesters (PBS, PBA, PBT) and copolyesters (PBST45 and PBAT45) predicted by group contribution method, Table S3: Data for crystallinity calculation of PBST and PBAT film, Figure S1: Theoretically predicted gas permeability coefficients of PBSTs and PBATs in full range of composition and crystallinity, Figure S2: ^1^H NMR spectra (solvent: CDCl_3_) of PBST and PBAT copolyesters (left) and peak assignment (right), Figure S3: First heating DSC curve of various PBST and PBAT copolyesters, Figure S4: Positron annihilation lifetime spectroscopy (PALS) of PBST44 and PBAT48 copolyesters. Figure S5: (A) Rapid cooling DSC curves of PBST44 (green) and PBAT48 (red) after erasing heat history; (B) Heat capacity vs. temperature cures around *T*_g_ of completely amorphous (blue) and semi-crystalline (pink, did not erase heat history) PBST44 samples; (C) Heat capacity vs. temperature cures around *T*_g_ of completely amorphous (orange) and semi-crystalline (black, did not erase heat history) PBAT48 samples.

## References

[1] Siegenthaler K.O., Kunkel A., Skupin G., Yamamoto M. (2012). Ecoflex^®^ and Ecovio^®^: Biodegradable, performance-enabling plastics. Adv. Polym. Sci..

[2] Huang F.F., Wu L.B., Li B.G. (2020). Sulfonated biodegradable PBAT copolyesters with improved gas barrier properties and excellent water dispersibility: From synthesis to structure-property. Polym. Degrad. Stab..

[3] Livi S., Sar G., Bugatti V., Espuche E., Duchet-Rumeau J. (2014). Synthesis and physical properties of new layered silicates based on ionic liquids: Improvement of thermal stability, mechanical behaviour and water permeability of PBAT nanocomposites. RSC Adv..

[4] Ren P.G., Liu X.H., Ren F., Zhong G.J., Ji X., Xu L. (2017). Biodegradable graphene oxide nanosheets/poly-(butylene adipate-co-terephthalate) nanocomposite film with enhanced gas and water vapor barrier properties. Polym. Test..

[5] Calderaro M.P., Sarantopóulos C.I.G.d.L., Sanchez E.M.S., Morales A.R. (2020). PBAT hybrid nanofillers composites-part 1: Oxygen and water vapor permeabilities, UV barrier and mechanical properties. J. Appl. Polym. Sci..

[6] Reis M.O., Zanela J., Olivato J., Garcia P.S., Yamashita F., Grossmann M.V.E. (2014). Microcrystalline cellulose as reinforcement in thermoplastic starch/poly(butylene adipate-co-terephthalate) films. J. Polym. Environ..

[7] Savadekar N.R., Kadam P.G., Mhaske S.T. (2013). Studies on the effect of nano-alumina on the performance properties of poly(butylene adipate-co-terephthalate) composite films. J. Thermoplast. Compos. Mater..

[8] Hu H., Zhang R.Y., Wang J.G., Ying W.B., Shi L., Yao C.K., Kong Z.Y., Wang K., Zhu J. (2019). A mild method to prepare high molecular weight poly(butylene furandicarboxylate-co-glycolate) copolyesters: Effects of the glycolate content on thermal, mechanical, and barrier properties and biodegradability. Green Chem..

[9] Falcão G.A.M., Vitorino M.B.C., Almeida T.G., Bardi M.A.G., Carvalho L.H., Canedo E.L. (2017). PBAT/organoclay composite films: Preparation and properties. Polym. Bull..

[10] Pan H., Hao Y., Zhao Y., Lang X., Zhang Y., Wang Z., Zhang H., Dong L. (2017). Improved mechanical properties, barrier properties and degradation behavior of poly(butylenes adipate-co-terephthalate)/poly(propylene carbonate) films. Korean J. Chem. Eng..

[11] Wang L.F., Rhim J.W., Hong S.I. (2016). Preparation of poly(lactide)/poly(butylene adipate-co-terephthalate) blend films using a solvent casting method and their food packaging application. LWT Food Sci. Technol..

[12] Benes H., Kredatusova J., Peter J., Livi S., Bujok S., Pavlova E., Hodan J., Abbrent S., Konefal M., Ecorchard P. (2019). Ionic liquids as delaminating agents of layered double hydroxide during in-situ synthesis of poly (butylene adipate-co-terephthalate) nanocomposites. Nanomaterials.

[13] Li J.X., Lai L., Wu L.B., Severtson S.J., Wang W.J. (2018). Enhancement of water vapor barrier properties of biodegradable poly(butylene adipate-co-terephthalate) films with highly oriented organomontmorillonite. ACS Sustain. Chem. Eng..

[14] Bechthold I., Bretz K., Kabasci S., Kopitzky R., Springer A. (2008). Succinic acid: A new platform chemical for biobased polymers from renewable resources. Chem. Eng. Technol..

[15] Song H., Lee S.Y. (2006). Production of succinic acid by bacterial fermentation. Enzym. Microb. Technol..

[16] Sun Y.J., Wu L.B., Li N.X., Dai J.M. (2016). Co-esterification process for synthesis of aliphatic-aromatic co-polyester poly(butylene succinate-co-butylene terephalate). Chem. React. Eng. Technol..

[17] Hu L.X., Wu L.B., Song F.C., Li B.G. (2010). Kinetics and modeling of melt polycondensation of PBST 1-esterification. Macromol. Reat. Eng..

[18] Liu T.Q., Gu X.G., Li N.X., Wu L.B., Wang J.J., Feng L.F. (2019). Modeling of coesterification process for biodegradable poly(butylene succinate-co-butylene terephthalate) copolyesters. Macromol. Reat. Eng..

[19] Li F.X., Xu X.X., Hao Q.H., Li Q.B., Yu J.Y., CAO A.M. (2006). Effects of comonomer sequential structure on thermal and crystallization behaviors of biodegradable poly(butylene succinate-co-butylene terephthalate)s. J. Polym. Sci. Pol. Phys..

[20] Lee S.H., Lim S.W., Lee K.H. (1999). Properties of potentially biodegradable copolyesters of (succinic acid-1,4-butanediol)/(dimethyl terephthalate-1,4-butanediol). Polym. Int..

[21] Sun Y.J., Wu L.B., Bu Z.Y., Li B.G., Li N.X., Dai J.M. (2014). Synthesis and thermomechanical and rheological properties of biodegradable long-chain branched poly(butylene succinate-cobutylene terephthalate) copolyesters. Ind. Eng. Chem. Res..

[22] Lu J., Wu L.B., Li B.G. (2017). Long chain branched poly(butylene succinate-co-terephthalate) copolyesters using pentaerythritol as branching agent: Synthesis, thermo-mechanical, and rheological properties. J. Appl. Polym. Sci..

[23] McKeen L.W. (2017). Permeability Properties of Plastics and Elastomers.

[24] Lagaron J.M., Catalá R., Gavara R. (2004). Structural characteristics defining high barrier properties in polymeric materials. Meter. Sci. Techol..

[25] Perkins W. (1988). Effect of molecular weight and annealing temperature on the oxygen barrier properties of oriented PET film. Polym. Bull..

[26] Vannini M., Marchese P., Celli A., Lorenzetti C. (2015). Fully biobased poly(propylene 2,5-furandicarboxylate) for packaging applications: Excellent barrier properties as a function of crystallinity. Green Chem..

[27] Salame M. (1986). Prediction of gas barrier properties of high polymers. Polym. Eng. Sci..

[28] Askadskii A.A., Afanas’ev E.S., Matseevich T.A., Popova M.N., Kovriga O.V., Kondrashchenko V.I. (2015). The calculation scheme for estimation of the water permeability through polymers and copolymers. Polym. Sci. Ser. A.

[29] Gan Z.G., Kuwabara K., Yamamoto M., Abe H., Doi Y. (2004). Solid-state structures and thermal properties of aliphatic-aromatic poly(butylene adipate-co-butylene terephthalate) copolyesters. Polym. Degrad. Stab..

[30] Nagata M., Goto H., Sakai W., Tsutsumi N. (2000). Synthesis and enzymatic degradation of poly(tetramethylene succinate) copolymers with terephthalic acid. Polymer.

[31] Zhu X.H., Cheng W., Zhu G.X., Lv J.L., Zhang Y.X., Zhang W. (2007). Structure and properties of poly(butylene terephthalate-co-butylene succinate) copolyesters synthesized with rare earth-titanium catalyst. Pet. Technol..

[32] Cheng X.R., Zhu G.X., Zhang W., Yan Y.F. (2012). Properties of biodegradable aliphatic-aromatic copolyesters. Pet. Technol..

[33] Zekriardehani S., Jabarin S.A., Gidley D.R., Coleman M.R. (2017). Effect of chain dynamics, crystallinity, and free volume on the barrier properties of poly(ethylene terephthalate) biaxially oriented films. Macromolecules.

[34] Duda J.L., Zielinski J.M. (1996). Free-Volume Theory. Diffusion in Polymers.

[35] Herrera R., Franco L., Rodríguez-Galán A., Puiggalí J. (2002). Characterization and degradation behavior of poly(butylene adipate-co-terephthalate)s. J. Polym. Sci. Pol. Chem..

[36] Lin J., Shenongin S., Nazarenko S. (2002). Oxygen solubility and specific volume of rigid amorphous fraction in semicrystalline poly(ethylene terephthalate). Polymers.

[37] Guinault A., Sollogoub C., Ducruet V., Domenek S. (2012). Impact of crystallinity of poly(lactide) on helium and oxygen barrier properties. Eur. Polym. J..

[38] Honga P.D., Chuang W.T., Yeh W.J., Lin T.L. (2002). Effect of rigid amorphous phase on glass transition behavior of poly(trimethylene terephthalate). Polymers.

[39] Mo Z.S., Cheng Y.Y. (1990). A Crystal-amorphous interphase in crystalline polymer. China Polym Bull..

