# Supplementary material for: Superior Gas Barrier Properties of Biodegradable PBST vs. PBAT Copolyesters: A Comparative Study
Source: Polymers (Basel). 2021 Oct 8;13(19):3449. doi: 10.3390/polym13193449 (PMC8512549; doi:10.3390/polym13193449)
Supplement: Supplementary file 1 [file polymers-13-03449-s001.zip › polymers-1410000-supplementary.pdf]

Superior Gas Barrier Properties of Biodegradable PBST vs. PBAT Copolyesters: A Comparative Study

Pengkai Qin [1,2], Linbo Wu [1,2,*], Bogeng Li [2], Naixiang Li [3], Xiaohu Pan [3] and Junming Dai [3]

1 Key Laboratory of Biomass Chemical Engineering of Ministry of Education, College of Chemical and Biological Engineering, Zhejiang University, 38 Zheda Road, Hangzhou 310027, China; qinpk@zju.edu.cn

2 State Key Laboratory of Chemical Engineering at ZJU, College of Chemical and Biological Engineering, Zhejiang University, 38 Zheda Road, Hangzhou 310027, China; bgli@zju.edu.cn

3 Sinopec Research Institute of Yizheng Chemical Fiber Co., Ltd., Puxi Road, Yizheng 211900, China; linx.yzhx@sinopec.com (N.L.); panxh.yzhx@sinopec.com (X.P.); daijm.yzhx@sinopec.com (J.D.)

* Correspondence: wulinbo@zju.edu.cn

Supporting information

Table S1. PBAT gas barrier data reported in literature.

Polymer	x_c [a]	P_{O2} [b]	P_{CO2} [b]	Test Conditions [c]	P_{WV} [d]	Test Conditions [c]	Source
Ecoflex		0.9					Ref. [1]
PBAT45		1.0	12.1	25/0	365	38/90	Ref. [2]
Ecoflex	9.3		8.3	20/0	285.4	30/53	Ref. [3]
Ecoflex	6.8	0.9		25/50	348.4	25/100	Ref. [4]
Ecoflex		1.0		23/0	333.5	38/90	Ref. [5]
Ecoflex					479.7	25/75	Ref. [6]
Ecoflex	10.6	1.2		30/50			Ref. [7]
Ecoflex		0.8	5.9	23/0	308.2	38/90	Ref. [8]
Ecoflex	11.5	1.2	13.7	25/0			Ref. [9]
Ecoflex	11.2	0.7	7.1	25/0			Ref. [10]
PBG7070	9.0				535.8	25/50	Ref. [11]
PBAT40	8.0	1.6	16.9	30/0			Ref. [12]

[a]: %. [b]: barrer (1barrer=10^{-10} $cm^3 \cdot cm/(cm^2 \cdot s \cdot cmHg)$). [c]: ^{o}C/ %RH. [d]: $g \cdot mm/(m^2 \cdot day \cdot atm)$.

Table S2. Gas permeability coefficients of some amorphous homopolyesters (PBS, PBA, PBT) and copolyesters (PBST45 and PBAT45) predicted by group contribution method.

Sample	P_{O2} (barrer) [a]	BIF_{O2} [b]	P_{CO2} (barrer) [a]	BIF_{CO2} [b]
PBA	1.39	1.0	6.26	1.0
PBS	0.85	1.6	3.68	1.7
PBT	0.29	4.8	1.15	5.4
PBAT45	0.69	1.0	2.92	1.0
PBST45	0.52	1.3	2.18	1.3

[a]: 1 barrer = 10^{-10} $cm^3 \cdot cm/(cm^2 \cdot s \cdot cmHg)$). [b]: Barrier improvement factor.

Table S3. Data for crystallinity calculation of PBST and PBAT film

Sample	ΔH_m (J/g) [a]	x_c (%) [b]	Source
PBS	-	60.0	Ref. [19]
PBST23	25.50	23.7	This work
PBST33	22.37	18.4	This work
PBST44	14.37	11.8	This work
PBST61	21.24	14.7	This work
PBST71	24.61	17.0	This work
PBT	-	40.0	Ref. [19]
PBAT48	11.44	10.0	This work

[a]: ΔH_m is the melting enthalpy calculated from the first heating curve. [b]: x_c (%) = $\Delta H_m/\Delta H^0_m$, ΔH^0_m is the melting enthalpy of 100% crystalline polymers and copolymers (with similar composition), ΔH^0_m of PBS, PBST23, PBST33, PBST44, PBST61, PBST71, PBT and PBAT48 are 110.5 J/g, 110.5 J/g, 121.4 J/g, 121.4 J/g, 144.5 J/g, 144.5 J/g, 144.5 J/g, 114 J/g, respectively, cited from ref. [4, 19]; x_c (crystallinity) of PBS and PBT are cited from ref. [19].

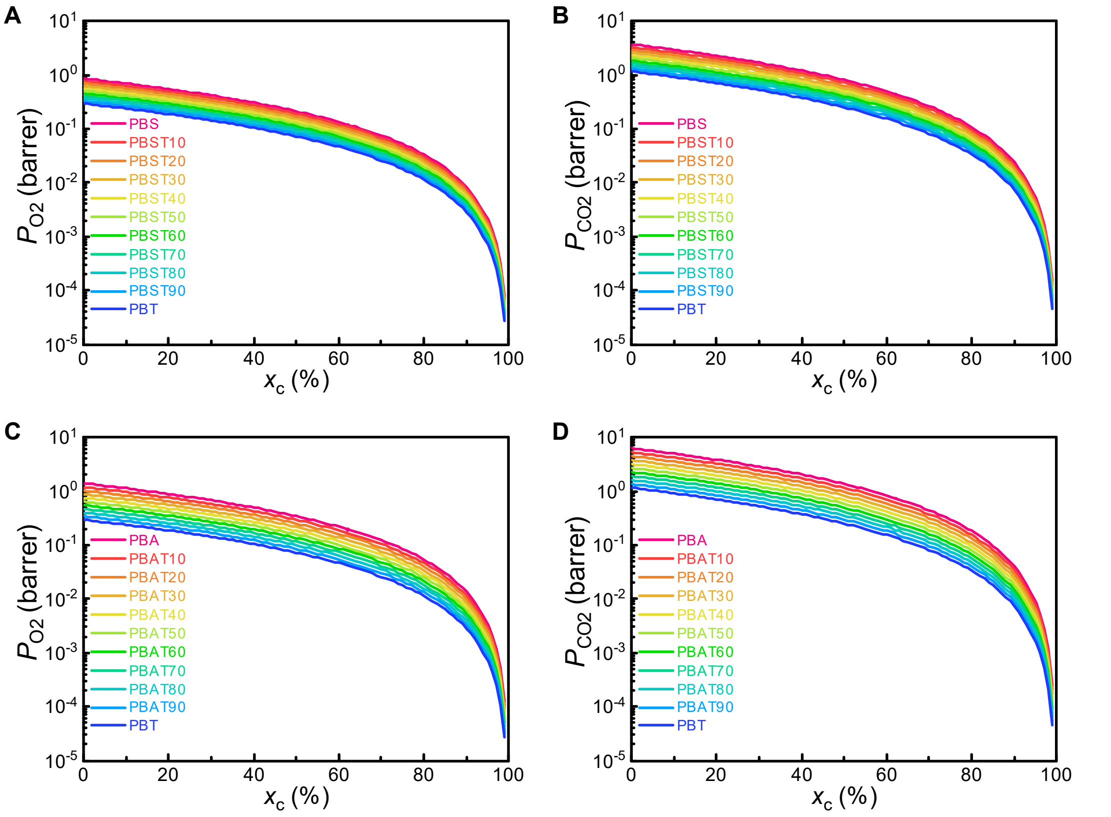

Figure S1. Theoretically predicted gas permeability coefficients of PBSTs and PBATs in full range of composition and crystallinity.

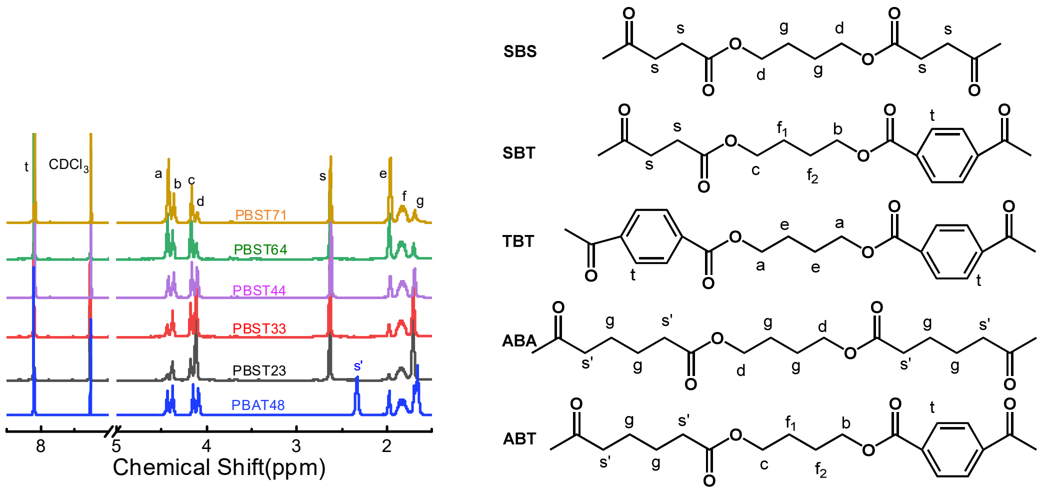

Figure S2. ^{1}H NMR spectra (solvent: $CDCl_3$) of PBST and PBAT copolyesters (left) and peak assignment (right).

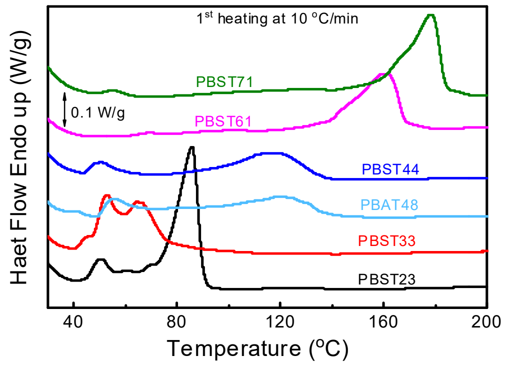

Figure S3. First heating DSC curve of various PBST and PBAT copolyesters.

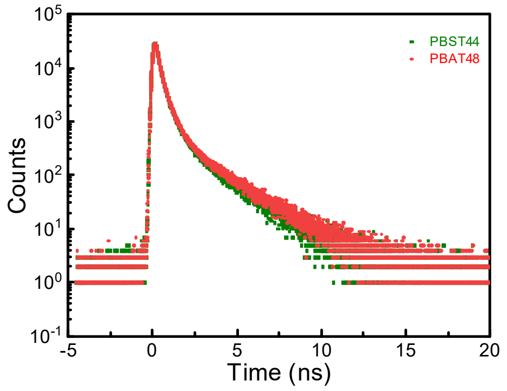

Figure S4. Positron annihilation lifetime spectroscopy (PALS) of PBST44 and PBAT48 copolyesters.

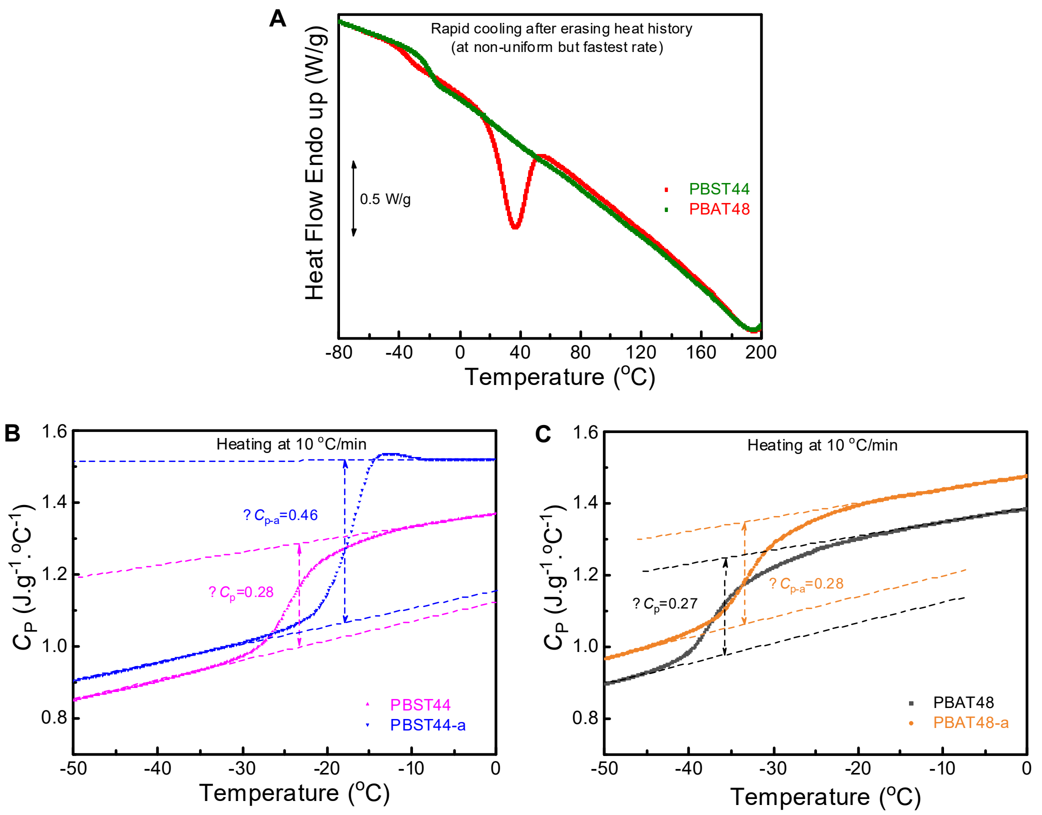

Figure S5. (A) Rapid cooling DSC curves of PBST44 (green) and PBAT48 (red) after erasing heat history; (B) Heat capacity vs. temperature cures around T_g of completely amorphous (blue) and semi-crystalline (pink, did not erase heat history) PBST44 samples; (C) Heat capacity vs. temperature cures around T_g of completely amorphous (orange) and semi-crystalline (black, did not erase heat history) PBAT48 samples.
